# An Apparatus for the Relief of Writers’ Cramp

**Published:** 1887-08

**Authors:** 


### An Apparatus for the Relief of Writer's Cramp.

Called the "Kaligraph" by its inventor, the late Mr. Charles Thurber. It consists of an iron frame-work, to which was attached a series of levers so arranged that by making large characters at one angle the characters were reproduced in ordinary size at the opposite angle. It was, in fact, a kind of reversed Pantagraph. Dr. Danna said that all writer's-cramp instruments were based on the principle of resting the groups of muscles most used, and throwing the work upon other groups. The Kaligraph fulfilled these indications better than any other instrument with which he was familiar. The objections to it were that it was cumbersome and expensive. The speaker showed cuts of all the various forms of instruments for writer's cramp (ten in all) which he had been able to collect. The kaligraph had been in practical use for thirty years, but it was very little known. It had enabled its inventor, who had suffered extremely from the cramp, to write with comfort. He was informed that Mr. Charles Dickens had possessed and used one.

Dr. G. S. Jacoby thought this instrument was only palliative, while Nussbaum's was also curative, and could be carried with one. It compelled the writer to use the abductors.

The President replied that an instrument calling into play another group of muscles of the hand would cause those to be affected after a time

Dr. Birdsall thought writer's cramp was due to cerebral fatigue rather than to muscular fatigue, and that instruments for overcoming it could be of only limited benefit.

---

There is no subject which has for the sanitarian more interest than that connected with the great mortality among the young children of our large cities. And as the principal factor in this mortality is represented by the term 'summer diarrhœa,' it is to diseases of this nature that especial attention is devoted by those who have at heart the welfare of the young. Thirty-five hundred persons succumbed to this class of diseases during the past year in New York City alone, more than half of the number in the two months of July and August. To diminish this mortality is a task worthy of the best efforts of the philanthropist; and every contribution to this end, however insignificant, should be gladly welcomed, and made, as far as it can be, the basis for action. Dr. L. Emmett Holt, of New York, in a paper recently read before the New York Academy of Medicine, has made a very valuable addition to our knowledge of the causes at work in the production of summer diarhœa, and to the methods for its treatment. After a full discussion of these points, he presents the following conclusions: 1. Summer diarrhœa is not to be regarded as a disease depending upon a single morbific agent. 2. The remote causes are many, and include heat, mode of feeding, surroundings, dentition, and many other factors. 3. The immediate cause is the putrefactive changes which take place in the stomach and bowels in food not digested, which changes are often begun outside the body. 4. These products may act as systemic poisons, or the particles may cause local irritation and inflammation of the intestine. In the treatment of the affection, Dr. Holt believes that antiseptics are of great value, especially naphthalin and the salts of salicylic acid.—*Science.*

Professor Gross states that he believes sub-iodide of bismuth is destined to replace, to a great extent, iodoform in the *antiseptic treatment of wounds.* It is being extensively used at the hospitals, and as yet, with none but most gratifying results.

The Writing and Printing of the Deranged.—The manuscripts of neuropaths—a word wide enough to include the slight and severe disturbances of mental sanity—present certain typical characteristics. They abound in italicized words; in exclamation points and punctuations after almost every word; in frequent use of capitals; in various sizes of writing; and the like. It is not often that such people have the opportunity of going to print and converting the compositor to their peculiar system of topography. M. Richet prints a few specimen pages of such an author, and counts twelve different kinds of letters in seventeen lines, besides the usual capitals, exclamation points, and so on, in great abundance. All this is significant of an excited, prancing state of mind closely allied to delirium and mania.—*Science.*

